# Long-Term Weight Outcomes after Bariatric Surgery: A Single Center Saudi Arabian Cohort Experience

**DOI:** 10.3390/jcm10214922

**Published:** 2021-10-25

**Authors:** Assim A. Alfadda, Mohammed Y. Al-Naami, Afshan Masood, Ruba Elawad, Arthur Isnani, Shaik Shaffi Ahamed, Nora A. Alfadda

**Affiliations:** 1 Obesity Research Center, College of Medicine, King Saud University, P.O. Box 2925 (98), Riyadh 11461, Saudi Arabia; afsmasood@ksu.edu.sa (A.M.); r.elawad@gmail.com (R.E.); aisnani@ksu.edu.sa (A.I.); 2 Department of Medicine, College of Medicine, King Saud University, P.O. Box 2925 (38), Riyadh 11461, Saudi Arabia; 3 Department of Surgery, College of Medicine, King Saud University, Riyadh 11461, Saudi Arabia; alnaami@ksu.edu.sa; 4 Department of Family and Community Medicine, College of Medicine, King Saud University, P.O. Box 7065, Riyadh 12372, Saudi Arabia; sshaik@ksu.edu.sa; 5 Clinical Nutrition Program, Department of Community Health Sciences, College of Applied Medical Sciences, King Saud University, P.O. Box 10219, Riyadh 11433, Saudi Arabia; noraalfadda@gmail.com

**Keywords:** weight regain, bariatric surgery, obesity, long-term follow-up, weight loss

## Abstract

Background: Obesity is considered a global chronic disease requiring weight management through lifestyle modification, pharmacotherapy, or weight loss surgery. The dramatic increase in patients with severe obesity in Saudi Arabia is paralleled with those undergoing bariatric surgery. Although known to be beneficial in the short term, the long-term impacts of surgery within this group and the sustainability of weight loss after surgery remains unclear. Objectives: We aimed to assess the long-term weight outcomes after bariatric surgery. Setting: The study was conducted at King Khalid University Hospital (KKUH), King Saud University Medical City (KSUMC) in Riyadh, Saudi Arabia. Methods: An observational prospective cohort study on adult patients with severe obesity undergoing bariatric surgery (sleeve gastrectomy (SG) or Roux-en Y gastric bypass (RYGB)) during the period between 2009 and 2015 was conducted. Weight loss patterns were evaluated pre- and post-surgery through clinical and anthropometric assessments. Absolute weight loss was determined, and outcome variables: percent excess weight loss (%EWL), percent total weight loss (%TWL), and percent weight regain (%WR), were calculated. Statistical analysis using univariate and multivariate general linear modelling was carried out. Results: A total of 91 (46 males and 45 females) patients were included in the study, with the majority belonging to the SG group. Significant weight reductions were observed at 1 and 3 years of follow-up (*p* < 0.001) from baseline. The %EWL and %TWL were at their maximum at 3 years (72.4% and 75.8%) and were comparable between the SG and RYGB. Decrements in %EWL and %TWL and increases in %WR were seen from 3 years onwards from bariatric surgery until the study period ended. The yearly follow-up attrition rate was 20.8% at 1 year post-surgery, 26.4% at year 2, 31.8% at year 3, 47.3% at year 4, 62.6% at year 5, and 79.1% at end of study period (at year 6). Conclusion: The major challenge to the successful outcome of bariatric surgery is in maintaining weight loss in the long-term and minimizing weight regain. Factors such as the type of surgery and gender need to be considered before and after surgery, with an emphasis on the need for long-term follow-up to enssure the optimal benefits from this intervention.

## 1. Introduction

Obesity has become a worldwide epidemic: according to the 2016 World Health Organization statistics, 1.9 billion adults were overweight, and more than 650 million were obese [1]. This increase in the number of individuals with overweight and obesity is also reflected in the Saudi population [2,3], where the estimated rate of overweight and obesity is 24.1% in men and 33.5% in women [3]. Obesity management aims at achieving weight loss, utilizing a multifactorial stepwise approach consisting of behavioral therapy, lifestyle and dietary interventions, and medical pharmacotherapy. Weight loss through bariatric surgery is an adjunct to the former strategies in patients who did not benefit from them or who have additional comorbidities associated with obesity. 

Presently, bariatric surgery is globally considered among the most effective management modalities for patients with obesity. The Saudi clinical practice guidelines, similar to the American Society for Metabolic and Bariatric Surgery, recommends that bariatric surgery should be conducted for patients with obesity who have a body mass index (BMI) ≥40 or ≥35 kg/m^2^ and the presence of comorbidities [4] to diminish the risk of the associated comorbidities and to improve quality of life [5,6]. In Saudi Arabia, the number of patients with obesity who undergo bariatric surgery has noticeably grown [7], and ≥15,000 procedures are estimated to be performed annually [8]. These procedures are known to be effective and lead to major weight loss, with the maximum weight loss occurring at 12–18 months post-surgery [9]. The Swedish Obese Subjects (SOS) study, the largest non-randomized intervention trial that compared weight loss outcome over 10 years, reported a maximal total body weight change in patients after 1 year after receiving Roux-en-Y gastric bypass (RYGB) and vertical banded gastroplasty surgeries, with reductions of 38 ± 7% and 26 ± 10%, respectively [5]. A successful outcome of bariatric surgery is one that achieves a loss of 50–70% of excess weight (EWL) or the 20–30% loss of the patient’s initial weight, or a BMI < 35 kg/m^2^ [9,10]. Although many studies have documented the competence and effectiveness of bariatric surgery in reducing excess weight in the short term, mid- and long-term studies have reported weight regain as an index for failure of the surgery, regardless of the surgical procedure [5,11]. According to the SOS study, patients were noted to have regained around 20–25% of their lost weight at 10 years post-surgery. A weight regain of 12% total body weight was observed in patients who underwent RYGB, while those reported for sleeve gastrectomy (SG) were variable, ranging from 6% at as early as two years post-surgery to 76% at six years post-surgery [12]. Previous studies have addressed different factors that contribute to weight regain and the failure of bariatric surgery, including dilation of the gastric pouch or gastro-jejunal anastomosis, pre-surgery BMI, eating behaviors, increases in energy intake, level of physical activity, the patient’s lack of commitment to follow-up visits, and psychological factors [13,14,15,16,17,18].

Obesity, similar to other chronic diseases, persists for prolonged durations and requires a continuous close follow-up to re-assess the efficacy of treatments, including bariatric surgeries. Compared to the number of surgeries being performed, there are few mid-long-term studies assessing the effectiveness and changes in weight loss. This is even more true in case of the Saudi population, where only a few studies have addressed bariatric surgery outcomes [4,7,8,19,20] and fewer have evaluated the long-term weight loss outcomes. In this study, we aim to observe and evaluate the weight evolution pattern in Saudi patients during a six-year follow-up period following bariatric surgery. 

## 2. Materials and Methods

### 2.1. Study Design, Setting and Subjects

An observational prospective cohort study of adult patients with obesity (age ≥ 18 years) who underwent SG and RYGB was conducted at King Khalid University Hospital (KKUH), King Saud University Medical City (KSUMC) in Riyadh, Saudi Arabia, between 2009 and 2015. All procedures and protocols were reviewed and approved by the Research Ethics Committee of the College of Medicine, King Saud University. Written informed consent was obtained from all participants. The bariatric procedures were conducted under the supervision of a single bariatric surgeon. Patients who underwent bariatric surgery and who had follow-up visits at the Obesity Clinic at KKUH and the Obesity Research Center for a period of 6 years were included in the study. Their pre and postoperative clinical data and anthropometric measurements were collected and recorded during follow-up. 

Weight (in kilograms) was measured in light clothing and without shoes to the nearest 0.1 kg. Height was measured using a stadiometer, and BMI was calculated. The variables analyzed were age, sex, BMI, absolute weight loss, %EWL, percent total weight loss (%TWL), and percent weight regain (%WR).

### 2.2. Calculated Variables

We calculated the absolute weight loss as ((follow-up weight − pre-surgery weight/pre-surgery weight) × 100) for all of the different time points. The outcome variables in the study included %EWL, %TWL, and %WR. The %EWL was calculated as ((pre-surgery weight − follow-up weight)/(operative excess weight)) × 100, where the operative excess weight equaled (pre-surgery weight − ideal weight) and where the ideal weight was based on the metropolitan tables [21]. A %EWL of ≥50% represented successful weight loss; a %EWL of ≤50% was considered as a failure [22]. In other studies, the rates indicating failure were reported to be ≤25% at ≥5 years [23,24]. Given the variability of %EWL depending on the definition of the ideal body weight, we used %TWL, as it is reported to be less influenced by BMI and other anthropometric measures. The %TWL was calculated as follows: ((preceding year weight − current weight)/preceding year weight) × 100 [25]. The %WR, which is the percentage of weight regained from the nadir weight (lowest measured post-surgery weight), was calculated using the following formula: ((current weight − nadir weight))/(pre-surgery weight − nadir weight) × 100), where ≥25% weight gain from nadir was considered to be excessive weight regain [22]. After surgery, the patients were prospectively followed up with in the clinic for 6 years. A comprehensive anthropometric measurement (weight, height, and BMI) was performed before surgery and post-surgery at each time point annually until the end of the study period. The yearly attrition rate (in %) was derived by dividing the number of withdrawn participants (calculated as the number of participants retained in the study subtracted from the total number of participants originally included in the study at pre-surgery) by the number of participants originally included in the study × 100 [26].

### 2.3. Statistical Analysis 

Data were analyzed using the SPSS 24.0 Advanced statistics module (IBM Inc., Chicago, IL, USA). Categorical variables (gender and type of surgery) were reported as numbers and percentages, whereas continuous variables (age, anthropometric measurements, %EWL, %TWL and %WR) were reported as mean, standard deviation, and range. The mean weights for all patients from pre-surgery and across the six follow-up time points were graphically presented according to gender and type of surgery. 

Changes in the mean values of three outcome variables: %EWL, %TWL, and %WR, over the six time points were compared using the repeated measures analysis of variance (ANOVA), and F values were reported to represent the systematic variance of %EWL, %TWL, and %WR across the six time points. Repeated measures of analysis for the outcome variables (%EWL, %TWL, and %WR) with each of the independent variables (gender and type of surgery) were also conducted.

A generalized linear mixed model for repeated measures for univariate and multivariate analysis were used to evaluate the changes in the quantitative outcome variables, which were observed at the six observation time points (1 to 6 years post-surgery). The fixed effects used in the model were time points, gender, and type of surgery. Akaike-corrected information criteria were used to identify the model of best fit. The least significant difference criterion was used to calculate the adjusted *p*-values in a pairwise comparison of the mean values. A *p*-value of <0.05 was used to report the statistically significant results.

## 3. Results

A total of 91 (50.5% male) patients with the mean age of 33.3 ± 9.7 years who underwent bariatric surgery were included in the study at baseline. The mean pre-surgery weight and BMI were 134.4 ± 33.8 kg and 49.7 ± 9.9 kg/m^2^, respectively. Table 1 shows a detailed demographic profile of the total study population. 

The overall annual follow-up rate of the patients was 79.1% (33 males/39 females) at 1 year, 81.3% (35 males/39 females) at 2 years, 68.1% (30 males/33 females) at 3 years, 54.9% (27 males and 23 females), 42.9% (17 males and 17 females), and 20.9% (7 males, 12 females) at 6 years. The yearly follow-up attrition rate was 20.8% at year 1 post-surgery, 26.4% at year 2, 31.8% at year 3, 47.3% at year 4, 62.6% at year 5, and 79.1% at end of the study period, i.e., year 6 post-surgery.

**Figure 1:**
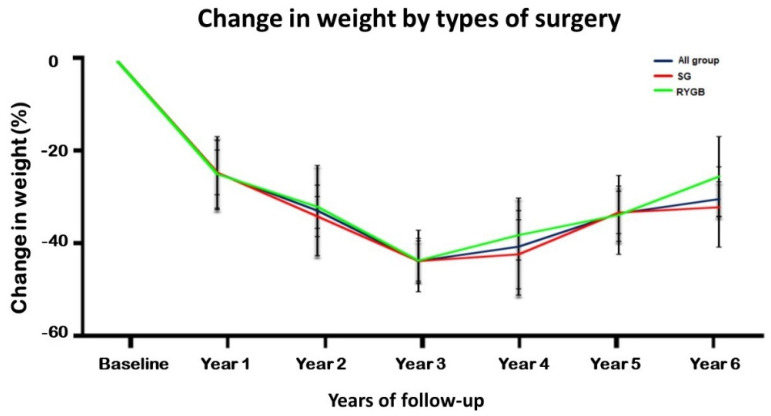
Graphical representations of changes in weight (%), with the error bars representing the ±2 SD during the 6-year follow-up period for all patients who underwent bariatric surgery (blue line), for those who underwent SG (red line), and those who underwent RYGB (green line).

To characterize the weight change patterns in our cohort, a subgrouping of the patients according to the type of surgery and gender was conducted. Based on the type of surgery, the patients in the RYGB group accounted for 31.9% of patients (*n* = 29, 11 males and 18 females) while the SG group comprised 68.1% of patients (*n* = 62, 35 males and 27 females). The maximum mean weight-loss percentage in the RYGB and SG groups was seen at 3 years post-surgery and was similar (54.3% and 54.4%, respectively). Increments in weight were observed in both the bariatric surgery groups beyond the 3-year follow-up period. Both the groups also showed an increasing weight gain trend from year 4 post-surgery and onwards (Figure 1).

**Figure 2:**
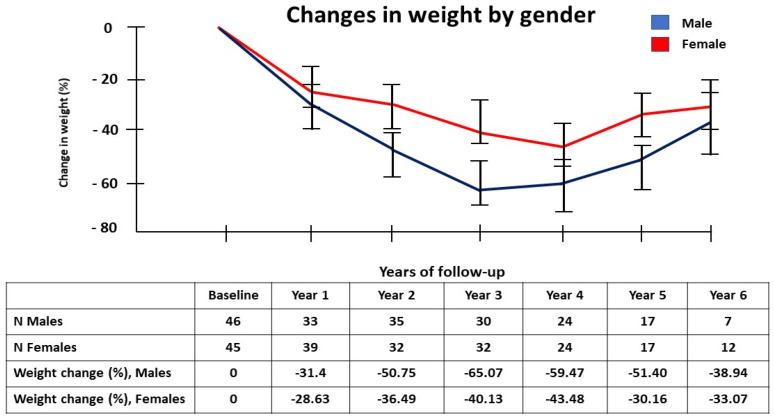
Graphical representation of weight change (%), with the error bars representing the ±2 SD, according to gender of the patients post bariatric surgery during the 6-year follow-up period. The number of patients at each time point and the mean percentage weight loss in male and female participants is shown. SD-standard deviation.

Based on gender, we noticed that the maximum mean percentage weight loss occurred at 3 years post-surgery by as much as −65.07% in males, while it was −43.48% at 4 years post-surgery for females (Figure 2). The rate of weight regain was seen to increase gradually from 3 years post-surgery onwards until the end of the study period. Significant weight regain (defined as ≥25% weight gain from nadir weight) was seen in 53.3% of the patients after 6 years. 

**Figure 3:**
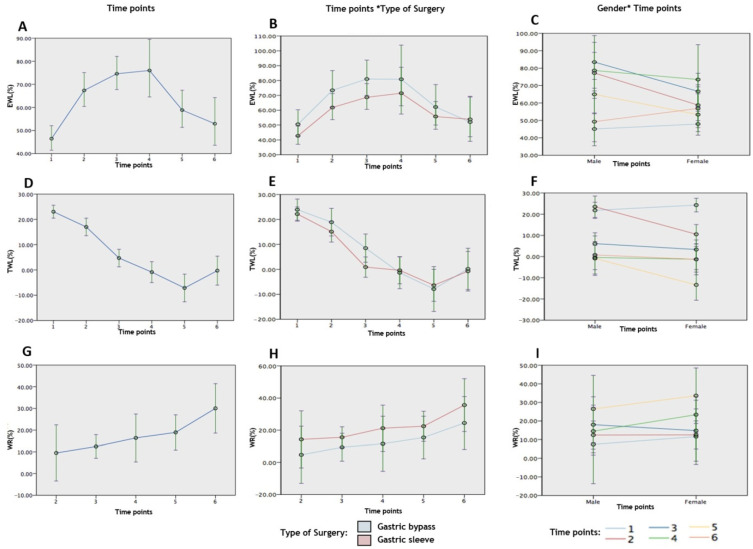
The figure shows a line graph with 95% confidence intervals and depicts the changes in the estimated mean of outcome variables with the predictors over the study duration at the different time points. The changes in %EWL with (**A**) different time points, (**B**) time points and type of surgery, and (**C**) time points and gender; the changes in %TWL with (**D**) different time points, (**E**) time points and type of surgery, and (**F**) time points and gender; and the changes in %WR with (**G**) different time points, (**H**) time points and type of surgery, and (**I**) time points and gender are shown.

Three outcome variables (%EWL, %TWL, and %WR) were considered for the analysis. A total of 314, 308, and 98 repeated measures were used in the analysis for these outcome variables. Table 2 shows the comparison of the mean values of %EWL, %TWL, and %WR across the six observation time points, and Table 3 shows the comparison of mean values of %EWL, %TWL, and % WR across the observation time points in male and female patients and in those who had undergone RYGB and SG surgeries. A similar comparison is shown for %EWL (Figure 3A–C), %TWL (Figure 3D–F), and %WR (Figure 3G–I).

### 3.1. Univariate Analysis: Generalized Linear Mixed Effects Model Analysis for the Outcome Variables %EWL, %TWL and %WR for Each of the Independent Variables (Time Points, Type of Surgery and Gender)

The univariate repeated measures and generalized linear mixed effects model for the outcome variables %EWL, %TWL, and % WR across the six time points showed statistically significant differences (F = 10.82, *p* < 0.0001; F = 43.99, *p* < 0.0001; F = 2.72; *p* = 0.034) (Table 2). 

The mean %EWL values were significantly increased at 3 and 4 years post-surgery when compared to the mean values at 6 years post-surgery (*p* < 0.0001), where the coefficients at 3 (19.04, *t* = 3.21, *p* = 0.001) and 4 (21.02, *t* = 2.74, *p* = 0.006) years indicate that %EWL increased by 19.04 and 21.02 units when compared to the mean value of %EWL at 6 years post-surgery. 

The mean %TWL values were significantly higher at 1 and 2 years post-surgery when compared to the mean values at 6 years post-surgery (*p* < 0.0001), where the coefficients at 1 (23.35, *t* = 7.90, *p* < 0.0001) and 2 years (16.57, *t* = 5.08, *p* < 0.001) showed that the %TWL mean values increased by 23.35 and 16.57 units when compared to the mean value of %TWL at 6 years post-surgery.

The mean %WR values were significantly decreased at 2 (−20.18, *t* = −2.67, *p* = 0.009) and 3 (−17.06, *t* = −2.85, *p* = 0.005) years post-surgery when compared to the mean values at 6 years post-surgery, indicating that the %WR mean values decreased by 20.18 and 17.06 units when compared to the mean value of %WR at 6 years post-surgery. 

The comparison of the mean values of %EWL in each of the surgery groups (RYGB and SG) across the six time points showed highly statistically significant differences (F = 2.89, *p* = 0.018 and F = 9.27, *p* < 0.0001), respectively (Table 3). The mean values of %EWL in patients who underwent RYGB surgery at 3 years was increased by 24.82 units (*t* = 2.24, *p* = 0.027), while it increased by 15.89 units (*t* = 2.14, *p* = 0.033) in those who underwent SG when compared to the mean value of %EWL at 6 years post-surgery. 

Similarly, high statistically significant difference was observed for the mean values of % TWL in each of the surgery groups (RYGB and SG) across the six time points (F = 20.53; *p* < 0.0001 and F = 27.63; *p* < 0.0001) (Table 3). The mean values of %TWL in patients who underwent RYGB surgery was higher at 1 and 2 years by 24.64 units (*t* = 4.85, *p* < 0.001) and 17.47 units (*t* = 3.19, *p* = 0.002), while it was higher by 22.87 units (*t* = 6.90, *p* < 0.001) and by 16.15 units (*t* = 4.24, *p* < 0.001) in those who underwent SG when compared to the mean value of %TWL at 6 years post-surgery. Hence, it can be inferred that the mean %TWL values were significantly lower after 6 years in patients who had undergone either surgery when compared to the mean values at 1 and 2 years post-surgery.

However, for the mean values of % WR, a non-significance was observed in each of the surgery groups (RYGB and SG) across the six time points (F = 2.28; *p* = 0.080 and F = 1.27, *p* = 0.294) (Table 3). However, the pairwise compassion of the time points shows that patients who underwent RYGB surgery had significantly decreased mean values of %WR at 2 and 3 years by −20.82 units (*t* = −2.64, *p* = 0.012) and 16.83 units (*t* = −2.24, *p* = 0.031), while those who underwent SG showed a significant decrease in the mean value of %WR at 3 years by 19.55 units (*t* = −2.10, *p* = 0.041) when compared to the mean value of %WR at 6 years post-surgery.

The comparison of the mean values of %EWL in each of the gender groups (male and female) across the six time points showed highly statistically significant differences (F = 7.22; *p* < 0.0001 and F = 4.74; *p* < 0.0001) (Table 3). The mean %EWL values in males were significantly increased at 2 (24.70, *t* = 2.45, *p* = 0.015), 3 (38.90, *t* = 10.18, *p* = 0.003), and 4 (26.61, *t* = 2.21, *p* = 0.029) years when compared to the mean value at 6 years post-surgery. In female subjects, the mean values of %EWL across the six time points were statistically significantly different, whereas the comparison of each time point with the mean values at 6 years post-surgery did not show any statistically significant differences. 

Additionally, highly statistically significant differences were observed for the mean values of % TWL in both male and female subjects across the six time points (F = 16.67; *p* < 0.0001 and F = 39.15; *p* < 0.0001) (Table 3). The pairwise comparison showed that the mean %TWL values in males was significantly higher at 1 (20.69, *t* = 5.75, *p* < 0.001) and at 2 (22.10, *t* = 5.23, *p* < 0.001) years when compared to the mean value at 6 years post-surgery. This was also noted in the female subjects, and the mean values of %TWL were found to be significantly higher at 1 (25.37, *t* = 4.38, *p* < 0.001) and 2 (11.53, *t* = 4.53, *p* = 0.012) years when compared to the mean values of %TWL at 6 years post-surgery.

In each of the gender groups (male and female) the comparison of the mean values of %WR across the five time points of observations showed no statistically significant differences (F = 1.54, *p* = 0.209 and F = 1.74, *p* = 0.156) (Table 3). However, the pairwise comparison of the time points indicated that the mean %WR values in males were significantly decreased at 2 years (−20.91, *t* = −2.34, *p* = 0.024) while in females, they were significantly decreased at 3 years (−18.39, *t* = 4–2.14, *p* = 0.037) when compared to the mean values of %WR at 6 years post-surgery (Figure S1I).

### 3.2. Multivariate Analysis: General Linear Mixed Effects Modelling for Each of the Outcome Variables, %EWL, %TWL and %WR with Independent Variables (Time Points, Type of Surgery and Gender)

The multivariate repeated-measures generalized linear mixed effects model for the outcome variables %EWL and %TWL was significant for the overall model (F = 4.49, dff = 17, *p* < 0.0001 and F = 14.78, dff = 17, *p* < 0.0001). Significance was also noted with time points (F = 10.70, *p* < 0.001 and F = 40.05, *p* < 0.0001), gender (F = 3.53, *p* = 0.068 and F = 7.35, *p* = 0.007) and type of surgery (F = 4.14, *p* = 0.043 and F = 1.160, *p* = 0.282), time points × gender (F = 2.05, *p* = 0.071 and F = 3.36, *p* = 0.006), and time points × type of surgery (F = 0.310, *p* = 0.91 and F = 0.695, *p* = 0.628) were used as predictors of the model. 

Taking the time point predictors into consideration, significant differences (*p* < 0.001) in %EWL were observed at 2 (15.06), 3 (22.12), and 4 (23.09) years and for %TWL at 1 (23.34) and 2 (17.30) years in comparison to the mean value at 6 years post-surgery. The %EWL mean values in males were observed to be statistically significant at 2 (27.97), 3 (34.15), and 4 years (29.20) when compared to the mean value at 6 years post-surgery. Males showed significantly higher %EWL at 2 years post-surgery by 25.81 units (*p* = 0.045) compared to the females, while no significant differences were noted for the coefficients for the other terms in the model. The %TWL mean values in males were observed to be significant at 1 (21.07) and 2 years (22.70) and for females at 1 (25.60) and 2 (11.85) years when compared to the mean value at 6 years post-surgery. Similar to %EWL, %TWL in males was significantly higher at 2 years post-surgery compared to females. 

Considering surgery as a predictor, %EWL was significantly higher in male subjects who had undergone RYGB at 2 (21.91), 3 (29.18) and 4 (28.59) years post-surgery, while no statistically significant differences were observed in female subjects and in subjects who had undergone SG. Mean values of %TWL were statistically significant at 1 and 2 years post-surgery in patients who had undergone RYGB (23.75 and 18.73 units) and in SG the group (22.93 and 15.87) when compared to the mean values of %TWL at 6 years post-surgery.

The multivariate analysis for the outcome variable %WR with time points, gender, and type of surgery as predictors showed no statistical significance for the overall model (F = 1.10, dff = 14, *p* = 0.370). Among all of the coefficients, at 3 years post-surgery, the %WR mean value decreased by 23.59 units (*p* = 0.044) when compared to the mean value of %WR at 6 years post-surgery, whereas the coefficients for other terms in the model were not statistically significant. For the time points predictor, significant differences were observed at 2 (−20.53) and 3 years (−17.56) post-surgery when compared to the mean value at 6 years post-surgery. 

No statistically significant differences in the %WR pattern were observed in male subjects in the comparison of the mean values of %WR between the pair of time points (2 to 6 years post-surgery), whereas in female subjects, the mean values of %WR at 2 (−22.02) and at 3 years (−21.08) were significantly decreased when compared to the mean value at 6 years. Statistically significant differences were observed at 3 years in patients who had undergone SG surgery, where the mean values of %WR decreased by −20.06 units when compared to the mean values at 6 years post-surgery, while no significant differences were observed in patients who had undergone RYGB surgery (Figure S1).

## 4. Discussion

Being a chronic disease, managing or treating severe obesity is challenging and requires constant close clinical and nutritional monitoring to reduce the recurrence of weight gain after weight loss. A large body of literature has shown that weight loss, even at a modest level of 5–10%, is beneficial and helps in the resolution of obesity associated comorbidities including, T2DM, hypertension, and fatty liver disease, among others, and in improving the overall quality of life [27,28,29]. Attaining a weight loss change of ≥5 and/or ≥10% of initial body weight has also been shown to be strongly correlated with a reduction in the risk of cardiovascular disease [30]. Surgical intervention techniques such as bariatric surgery were instated to provide a more permanent weight loss solution. Regardless of the type of surgery or the mechanisms by which weight loss is achieved, bariatric surgery has proven to lead to a significant amount of weight loss immediately post-surgery [31], but its long-term efficacy needs to be evaluated. The general metrics to assess the success of the surgeries includes calculating %EWL (>50%), %TWL, and %WR post-surgery [32,33,34,35,36]. Different studies have shown a large amount of variability within these values, which have been attributed to either the type of surgery, the preoperative BMI, and to the race and ethnicity that the patients belong to [29,30]. To date, only a limited number of studies have looked at differences in weight loss patterns across different populations and specifically in the Saudi population, where bariatric procedures are presently being performed routinely. In our present study, we conducted a longitudinal follow-up of patients undergoing bariatric surgery and observed the weight evolution patterns through annual follow ups for a period of 6 years post-surgery.

### 4.1. Weight Evolution in the Overall Bariatric Group

All of the patients demonstrated a uniform annual incremental increase in the weight loss outcome measures (i.e., absolute weight change, %EWL, and %TWL) as early as 1 year post-surgery, and significant changes in these parameters were seen from baseline to up to 3 years post-surgery. Our findings are different from those of the previous studies that have reported maximum weight loss to occur at 1–2 years post-surgery. The %EWL and %TWL showed a similar pattern across the cohort, where incremental increases in weight loss were seen from the baseline, peaking at the 3-year post surgery. The calculated %EWL values observed in our study population were higher than those reported by Bohdjalian et al. at the 3-year annual follow up, and while long-term weight outcomes were similar to theirs [37], they were higher than those observed in other cohorts [37,38]. Weight change patterns beyond 3 years showed a decrease in the propensity for further weight loss followed by a plateauing phase and then a slow and gradual increase in the patients’ weight. Our findings are in line with the findings of the SM-BOSS study that also showed an increased BMI at 5 years [39].

Weight regain remains a major challenge in relation to the long-term success of bariatric surgery [40]. Numerous studies have previously shown a higher tendency for patients to regain their weight after an initial impressive weight loss until the midterm (>3 years), which was not substantiated in the long term (>5 years). Although weight regain is a consistent finding among studies, there are considerable inter-individual variations in the magnitude and rate of weight regain depending on factors ranging from behavioral, dietary, lifestyle, psychological, ethnic, and racial differences [22,40,41,42,43]. One of the reasons for the weight regain has been attributed to the influences of gastrointestinal hormones, including glucagon-like peptide 1 (GLP-1) ghrelin, glucose-dependent insulinotropic polypeptide (GIP), and the adipokine leptin. These hormones have been shown to regulate feelings of satiety, influence hunger and energy balance by regulating the intake and storage, and energy expenditure through the actions of the entero–hypothalamic axis [44,45]. In our study, we observed a gradual and consistent increase in the number of patients who experienced weight regain across the follow-up period. Significant weight regain (defined as ≥25% weight gain from nadir weight) was seen in 53.3% of the patients at 6 years post-surgery. The %WR was significantly higher in the 6th year in comparison to the 2nd and 3rd years of follow-up post-surgery. Our findings indicate that, similar to lifestyle and medical management of obesity, bariatric surgery is also successful in yielding short term weight loss. The notion that bariatric surgery provides a permanent solution for resolution in the long term has to be made with caution.

### 4.2. Weight Loss Patterns between SG and RYGB

The two most common weight loss surgical procedures that are performed are SG and RYGB, which differ in terms of the irreversible anatomical alterations created surgically at specific sites in the gastrointestinal tract (GIT). These anatomical changes in the GIT lead to many physiological and biochemical variations that produce differences in the regulation of the food and appetite, gut hormones, bile acids, and gut microbiota and consequently lead to weight loss by reducing appetite [46,47,48,49]. Few studies have claimed better weight loss outcomes with RYGB, while another study that conducted a head to head comparison between these two techniques suggests that in the long-term, only a subtle weight loss difference exists in favor of RYGB [49]. In our study, we found that the trajectories of weight loss in both the RYGB and SG demonstrated a similar trend when measured in terms of changes in weight % and %EWL from baseline up to 3 years post-surgery, at which point, the weight loss started decreasing. On the other hand, %TWL showed a steeper decline in weight loss in the RYGB group up until 4 years post-surgery compared to the SG group. Beyond the 4-year mark, both surgeries showed a similar weight pattern. Compared to SG, RYGB is considered to be the intervention that results in far greater weight loss. Our findings indicate that this assumption holds true with regard to weight loos in the short term, but the effects of both surgical procedures are similar in the long term. Our results are in line with the SLEEVEPASS and the SM-BOSS studies, which also reported no significant differences between the two bariatric methods with regard to weight loss in both the short or long term [48].

Previous studies have shown that on average, patients regain 7% of their total body weight from their lowest post-operative weight over the course of 10 years [50]. This weight regain pattern in our study was similar between the SG and RYGB groups. The RYGB group demonstrated an increase in the %WR in the 3-year post-surgery follow up, while weight regain in the SG group was seen from the 4-year follow-up onwards. Previously, the weight regain in patients who had undergone RYGB, was shown to be about 15% within two years of the surgery, which subsequently increased to 70% of patients between two and five years and to 85% at after five years post-surgery [51]. However, in our study, significant weight regain was noted at 3-year post-surgery follow up. The causes for weight regain have been shown to be due to homeostatic changes in the body post-surgery that lead to biochemical, physiological, hormonal, and metabolic adaptations to weight loss that support weight regain. These changes include perturbations in the levels of circulating appetite-related hormones and energy homoeostasis. In addition, the alterations in nutrient metabolism and subjective appetite are rather dependent on factors that depend on the physiology of the body and the metabolism. RYGB and SG induce similar changes in leptin, PYY, and GLP-1 levels, but not in the levels of ghrelin, whose levels are reduced after SG, while they are known to change over time after RYGB and may be the reason for the differences in weight regain between these methods [52]. The high prevalence of weight regain after bariatric surgery has also been a reason for an increase in the number of revisional bariatric surgeries that pose an increased surgical risk to the patient [53].

### 4.3. Weight Loss Changes According to Gender

In our study we also looked at the pattern of change in the weight loss patterns among the male and female participants at the different post-surgery time points. We found that males lost significantly more weight in terms of the mean values of %EWL in the 2, 3, and 4-year post-surgery follow ups, while the weight loss in the female subjects at each time point did not show any statistically significant differences with mean value of %EWL 6 years post-surgery. On the other hand, the mean %TWL values in both males and females were significantly higher at 1 and at 2 years post-surgery when compared to the mean values of %TWL at 6 years post-surgery. The differences in weight loss patterns among the genders were also seen in previous report by Tymitz et al., who showed that men had a higher absolute weight loss [54] while females showed a greater BMI loss post bariatric surgery [55]. This difference between the genders has been suggested to be the result of higher loss percentage of fat mass and an increase in fat free mass in men [56]. With regard to weight regain, we observed that males started to show an increase in weight earlier than their female counterparts, i.e., at the 3 years post-surgery. On the other hand, the weight regain seen in the females started at 4 years post-surgery, but both groups showed the same pattern at 6 years post-surgery. In their study, Meguid et al. attributed higher weight regain in females to differences in eating habits, diet, and probably a failure in developing and sustaining a large amount of plasma peptide YY levels, a hormone that regulates satiety and suppresses hunger [57]. The differences in the weight regain pattern of the males compared to the females within the two groups highlights the fact that gender is an important factor that affects the outcome of these surgeries. Additional studies to study the changes in the levels of these hormones in relation to differences in gender as well as with the type of surgery will be conducted in the future. Emphasis should be placed on regular follow ups post-surgery along with the implementation of a multidisciplinary approach to track weight regain to provide the best outcomes. 

To our knowledge, the present study is the first to have looked at the long-term weight changes that occur post bariatric surgery in patients with obesity from Saudi Arabia. As previously mentioned, a lack of standardization in the reporting measures used for weight loss outcomes has been noted in the literature, which has hindered direct comparisons of weight loss among various studies. Therefore, we used two weight loss measures or outcome variables, EWL% and TWL%, in the present study to provide a broader representation of the measures of weight loss that will allow our results to be more easily compared to those of other studies [32,33,58]. Our study has a number of limitations, specifically with regard to the attrition rate and number of patients that followed up, even after being given clinical appointments. One of the reasons for this could be the increased weight regain due to unhealthy dietary habits and behavioral lifestyle practices [17], which could have deterred the patients from attending their follow-up appointments. Although our follow-up rate is low, it is similar to that found by other groups attempting long-term follow-up studies [33,59,60]. All of the patients undergoing bariatric surgery were included without any prior stratification of their preoperative weight that could have affected the results.

## 5. Conclusions

The weight loss results that occur after bariatric surgery can be considered profound, as seen at 3 years post-surgery, but they are not consistent in the long run. Weight regain remains a major challenge post bariatric surgery, and long-term follow-up is required to ensure gaining the optimal benefits from this intervention. 

## Figures and Tables

**Table 1:** Overall demographic data and baseline characteristics of patients with obesity who underwent bariatric surgery. The values are represented as mean ± SD (standard deviation).

Demographic Variables	
Gender	
Male Female	46 (50.5%) 45 (49.5%)
Type of Surgery	
Sleeve Gastrectomy Roux-en-Y Gastric Bypass	62 (68.1%) 29 (31.9%)
Age at baseline, in years	
Mean ± SD Range	33.3 ± 9.7 17–60
Height, in meters	
Mean ± SD Range	1.64 ± 0.1 1.45–1.90
Pre-surgery weight, in kilograms	
Mean ± SD Range	134.4 ± 33.8 78.1–300.7
Pre-surgery BMI, in kg/m^2^	
Mean ± SD Range	49.7 ± 9.9 29.4–83.3

**Table 2:** Comparison of repeated measure (%EWL, %TWL, and %WR) mean (SD) values of study subjects across the six time points and the difference between each time point and at the end observation time point.

	Time Points (in Years)						F-Value	*p*-Value
Outcome Variables	1st	2nd	3rd	4th	5th	6th		
%EWL	45.46 (21.9)	65.71 (32.0)	73.14 (28.8)	75.12 (43.4)	58.04 (22.5)	54.11 (20.3)	10.82	<0.0001
%TWL	22.87 (10.0)	16.07 (15.6)	3.47 (13.2)	−0.73 (14.1)	−7.08 (16.4)	−0.49 (12.1)	43.99	<0.0001
%WR	–	10.20 (12.9)	13.32 (12.1)	17.58 (29.1)	20.81 (18.7)	30.38 (20.9)	2.72	0.034

%EWL—percentage excess weight loss, %TWL—percentage total weight loss, %WR—percentage weight regain.

**Table 3:** Comparison of repeated measure (%EWL, %TWL, and %WR) mean values across the six time points in male and female subjects and in relation to type of surgery.

Outcome Variables, Type of Surgery and Gender		Time Points (in Years)						F-Value	*p*-Value
		1st	2nd	3rd	4th	5th	6th		
%EWL	RYGB	51.21 (21.0)	71.18 (40.2)	79.0 (41.1)	80.04 (52.8)	60.7 (23.1)	54.20 (20.3)	2.89	0.018
	SG	42.64 (21.9)	62.80 (26.7)	69.91 (18.8)	72.20 (37.3)	56.78 (22.6)	54.02 (21.5)	9.27	<0.0001
%TWL	RYGB	24.41 (9.1)	17.23 (14.4)	8.04 (14.1)	−1.43 (15.6)	−9.96 (13.1)	−0.23 (14.9)	20.53	<0.0001
	SG	22.12 (10.5)	15.4 (16.4)	1.01 (12.1)	−0.24 (13.2)	−5.58 (18.0)	−0.755 (9.3)	27.63	<0.0001
WR	RYGB	–	5.42 (6.1)	9.41 (7.5)	11.31 (12.5)	17.91 (16.2)	26.25 (19.9)	2.28	0.080
	SG	–	14.97 (17.6)	15.55 (13.8)	21.76 (36)	22.44 (20.2)	35.1 (22.9)	1.27	0.294
%EWL	Male	42.96 (24.1)	74.34 (38.2)	80.54 (36.1)	76.26 (48.1)	63.15 (26.3)	49.64 (20.5)	7.22	<0.0001
	Female	47.52 (19.9)	58.15 (23.3)	66.21 (17.3)	73.84 (38.4)	52.93 (17.1)	56.71 (20.7)	4.74	<0.0001
%TWL	Male	21.32 (10.8)	22.72 (17.0)	4.20 (15.5)	−0.23 (17.9)	−0.51 (14.2)	0.62 (8.6)	16.67	<0.0001
	Female	24.13 (9.2)	10.29 (11.7)	2.84 (10.9)	−1.34 (7.5)	−13.3 (16.3)	−1.24 (14.3)	39.15	<0.0001
%WR	Male	–	7.42 (7.1)	13.28 (13.4)	20.0 (37.4)	16.61 (13.4)	28.33 (18.0)	1.54	0.209
	Female	–	11.59 (15.9)	13.36 (11.3)	14.42 (12.8)	23.62 (21.4)	31.75 (23.6)	1.74	0.156

## Data Availability

All data generated or analyzed in the current study are included in this article.

## Supplementary Materials

The following are available online at https://www.mdpi.com/article/10.3390/jcm10214922/s1, Figure S1: The figure shows a bar graph with 95% confidence intervals depicting changes in the outcome variables with the predictors over the study duration at the different time points. The changes in %EWL with (A) different time points, (B) time points and type of surgery, and (C) time points and gender are shown. The changes in %TWL with (D) different time points, (E) time points and type of surgery, and (F) time points and gender are shown. The changes in %WR with (G) different time points, (H) time points and type of surgery, and (I) time points and gender are shown. The horizontal line represents the estimated mean of the outcome variables at time point = 6. The vertical bars are the simple contrasts of the outcome variables at each level of time points minus the outcome variable at time point = 6. Significant contrasts are shaded in gold. The least significant difference adjusted significance level is 0.05.
