# Supplementary figures and images for: Long-Term Weight Outcomes after Bariatric Surgery: A Single Center Saudi Arabian Cohort Experience
Source: J Clin Med. 2021 Oct 25;10(21):4922. doi: 10.3390/jcm10214922 (PMC8584307; doi:10.3390/jcm10214922)

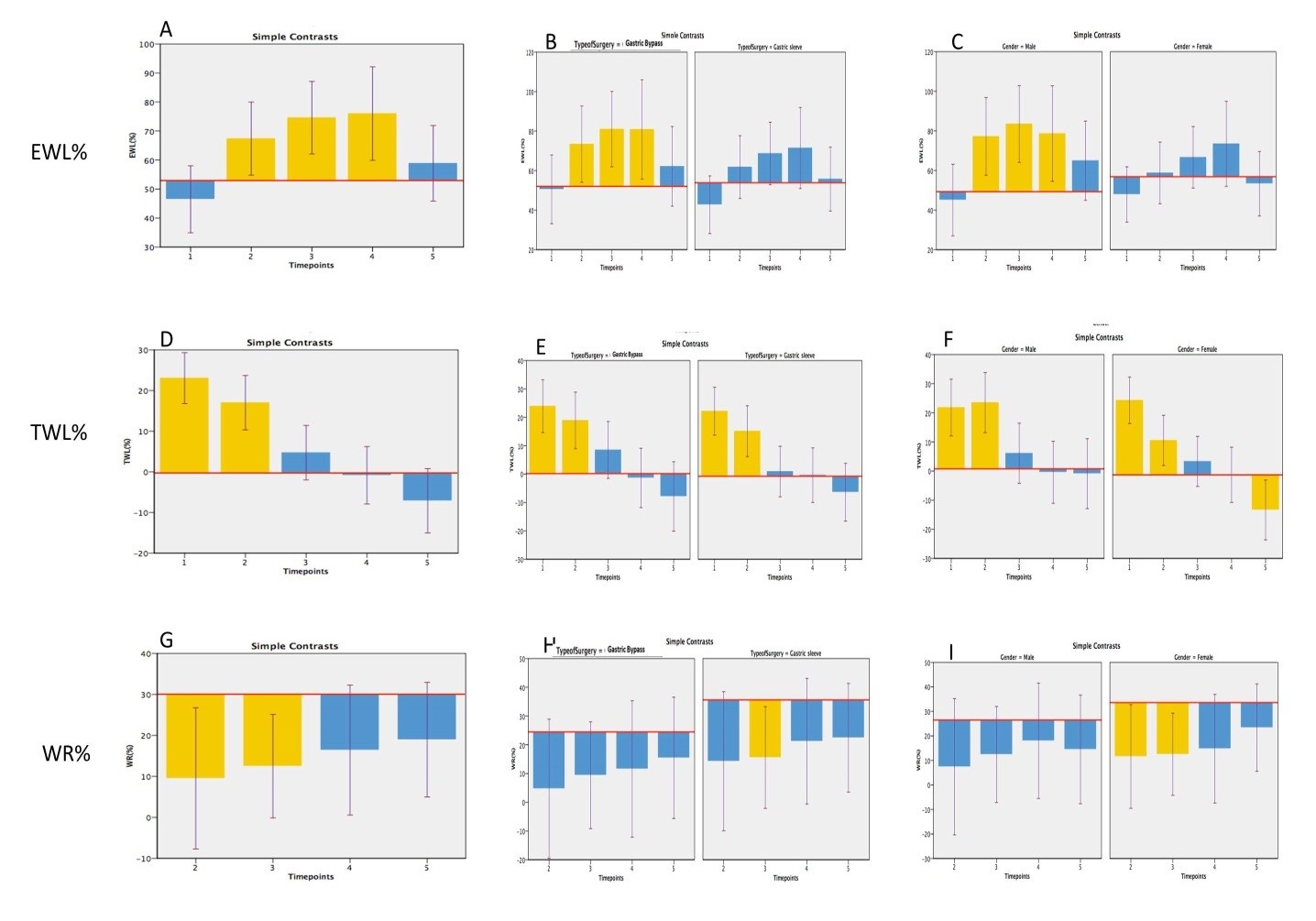

Supplement: Supplementary file 1 [file jcm-10-04922-s001.zip › jcm-1439236-supplementary.jpg]
